# 4D Self‐Morphing Culture Substrate for Modulating Cell Differentiation

**DOI:** 10.1002/advs.201902403

**Published:** 2020-02-18

**Authors:** Shida Miao, Haitao Cui, Timothy Esworthy, Bhushan Mahadik, Se‐jun Lee, Xuan Zhou, Sung Yun Hann, John P. Fisher, Lijie Grace Zhang

**Affiliations:** ^1^ Department of Mechanical and Aerospace Engineering The George Washington University 3590 Science and Engineering Hall, 800 22nd Street NW Washington DC 20052 USA; ^2^ Fischell Department of Bioengineering University of Maryland 3238 Jeong H. Kim Engineering Building College Park MD 20742 USA; ^3^ Department of Electrical and Computer Engineering Department of Medicine Department of Biomedical Engineering The George Washington University Washington DC 20052 USA

**Keywords:** 4D culture substrates, cell differentiation, neural regeneration, programmable culture substrates, regenerative medicine, stem cells

## Abstract

As the most versatile and promising cell source, stem cells have been studied in regenerative medicine for two decades. Currently available culturing techniques utilize a 2D or 3D microenvironment for supporting the growth and proliferation of stem cells. However, these culture systems fail to fully reflect the supportive biological environment in which stem cells reside in vivo, which contain dynamic biophysical growth cues. Herein, a 4D programmable culture substrate with a self‐morphing capability is presented as a means to enhance dynamic cell growth and induce differentiation of stem cells. To function as a model system, a 4D neural culture substrate is fabricated using a combination of printing and imprinting techniques keyed to the different biological features of neural stem cells (NSCs) at different differentiation stages. Results show the 4D culture substrate demonstrates a time‐dependent self‐morphing process that plays an essential role in regulating NSC behaviors in a spatiotemporal manner and enhances neural differentiation of NSCs along with significant axonal alignment. This study of a customized, dynamic substrate revolutionizes current stem cell therapies, and can further have a far‐reaching impact on improving tissue regeneration and mimicking specific disease progression, as well as other impacts on materials and life science research.

## Graphical abstract

A 4D programmable culture substrate with self‐morphing ability is presented to enhance dynamic growth and induce differentiation of stem cells. The result indicates the great potential of 4D culture substrates to serve as a new generation of commercially available scaffolds and devices that can be used to manipulate cell functions and fates.

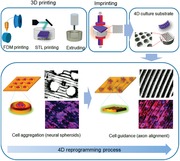

## 1 Introduction

The dynamic nature of the physiological environment in vivo plays an essential role in modulating cellular behaviors and functions, which is closely related to embryonic development, tissue self‐renewal or regeneration, wound healing, and disease progression over time.^[^[qv: 1–3]^]^ Stem cell therapy has provided a new paradigm for treating various diseases and tissue regeneration; however, these therapies have limited clinical application due to poor cell survival and differentiation potentials of the stem cells.^[^[qv: 1,4]^]^ Dynamic biochemical, physicochemical, and mechanostructural changes within the cellular microenvironment regulate many important biological functions of stem cells, including cell adhesion, growth, migration, and differentiation.^[^[qv: 2,3,5]^]^ Biochemical (bioactive molecules), mechanobiological (elasticity or force loads), and topographic cues have been extensively studied in vitro for their influence on stem cell behaviors. However, most efforts are centered on static patterns.^[^[qv: 6,7]^]^ Inspired by dynamic, tissue‐specific microenvironments in vivo, the use of dynamic cell culture platforms to create synthetic microenvironments in vitro has become an attractive means to direct active changes in cellular functionality and to replicate the dynamic complexity of native tissues.^[^[qv: 8,9,10]^]^

Several stimuli‐responsive methods, including applied electrical and magnetic fields, changes in pH and temperature, introduction of light and enzymes, have been applied to modulate the physicochemical properties of culture substrates.^[^[qv: 2,3,9,11,12]^]^ Specifically, these methods can be used to modify culture substrate surface properties (e.g., hydrophilicity/hydrophobicity), mechanical properties (e.g., elasticity), and scaffold topographies.^[^[qv: 2,9,10,12,13]^]^ However, there are few examples that present controlled, simultaneous regulation of these biologically relevant cues in order to influence stem cells at the more complex tissue level. Namely, those systems are simplified into single differentiation behaviors without cell–cell interactions, as compared to the complicated progression of stem cell development.^[^[qv: 2,10]^]^ As such, new developments in the dynamic regulation of the complex behaviors of stem cells with spatiotemporal controllability will be essential in developing novel dynamic biological tissue systems, and crucial for the full realization of their diverse applications.

Characteristically, stem cell‐based neural tissue engineering is an advantageous therapeutic strategy for treating neurodegenerative diseases and injuries.^[^[qv: 14]^]^ As such, neural tissue constructs can not only replace lost cells and secrete neurotrophic factors to stimulate axonal growth, but can also be genetically manipulated preimplantation, and can thereby repair severed axons and effectively restore locomotor functionality.^[^[qv: 15]^]^ In the developing central nervous system, neural stem cells (NSCs) dynamically mature following predetermined spatiotemporal developmental programs, and their biological characteristics greatly vary depending on the developmental stage considered.^[^[qv: 16,17]^]^ It has been observed that the in vitro differentiation of NSCs typically undergoes a critical morphological changing process (NSCs monolayer → aggregation → outmigration → differentiated NSCs with neurites) over a two‐week differentiation period.^[^[qv: 16]^]^ 3D cell aggregates can closely resemble the native configuration of NSCs in vivo, which allows for direct cell–cell signaling and cell–matrix interactions when compared to 2D culture substrates or 3D scaffolds.^[^[qv: 7,18]^]^ Several studies have induced NSC aggregation using a cell spheroid technique. However, the generation of conventional cell spheroids typically involves a complicated culturing process, which has been criticized for being sensitive to cell culture conditions, and often results in inconsistently differentiated cells, even between experimental trials.^[^[qv: 19,20]^]^ Moreover, the cell aggregation achieved using these methods largely leads to ineffective induction of differentiation, which fails to replicate the morphological and physiological features of NSCs at different developmental stages.^[^[qv: 20,21]^]^

To address this challenge, we propose an integrated strategy which combines printing and imprinting to create a more complex change in the cellular microenvironment, where 4D spatiotemporal cues are able to replicate the topographical and mechanobiological environment of native NSCs. 4D fabrication is a highly innovative, next generational additive manufacturing process which can be used to fabricate predesigned self‐assembly structures with a time‐dependent dynamic shape change.^[^[qv: 22,23]^]^ Within the diverse 4D mechanisms, shape memory polymers (SMPs) have attracted particular attention owing to their reversible “temporary‐permanent” thermomechanical reprogramming characteristics.^[^[qv: 24,25]^]^ Based on our previous experience in 4D fabrication,^[^[qv: 24,26,27]^]^ it was expected that the addition of the 4th dimension (time) would benefit the field of neural tissue engineering. This is because the dynamic 4D effect may better mimic the unique differentiation microenvironments of neural tissue and provide a potential method for recreating the different neurodevelopmental stages undergone by NSCs.

**Figure 1:**
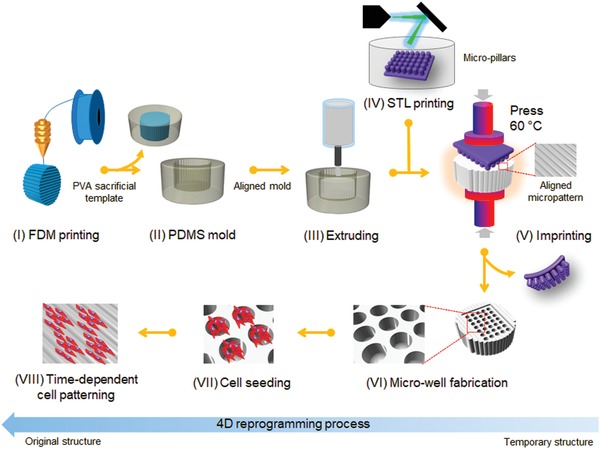
Schematic illustration of the fabrication procedure of a novel smart 4D neural substrate or scaffold with a time‐dependent topographic transformation, which is used to provide a new platform for modulating desired extracellular microenvironments for NSCs development at different differentiation stages (from NSC aggregation at an early stage to highly aligned micropatterns).

With the objective to control and manipulate the differentiation behaviors and functions of stem cells, we designed and fabricated a novel 4D substrate as a new culture platform for providing desirable dynamic extracellular microenvironments. As a model, a 4D neural culture substrate was developed to replicate NSC and neural cell development at different stages of differentiation (from NSC aggregation at an early stage to highly aligned micropatterns), as illustrated in **Figure** **1**. To the best of our knowledge, we are the first to use a smart 4D culture substrate with a time‐dependent complex topographic transformation to replicate the dynamic process of neural development. The 4D culture system was not only able to create a unique, dynamic 3D pattern to precisely control cell–cell interactions and differentiation behaviors for stem cell biological study, but also could be integrated with biomanufacturing techniques to generate a dynamic physiological environment to induce stem cell‐based tissue regeneration.

## 2 Results and Discussion

### 2.1 Preparation and Optimization of Shape Memory Polymers

**Figure 2:**
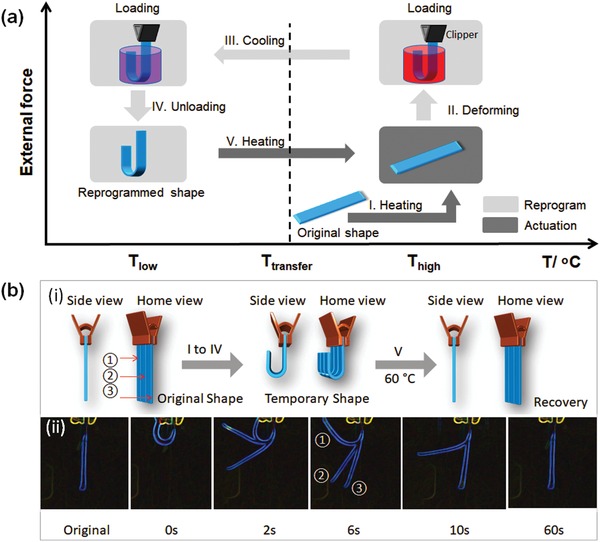
Shape memory properties of the synthesized polymeric materials. a) Illustration of the process of shape memory effect. I, increase the temperature over transition temperature; II, exert a U shape change with enforced restriction; III, fix a temporary U shape at a lower temperature; IV, remove externally enforced restriction; V, increase the temperature to recover the original shape. b) Shape recovery of the synthesized materials. ① BP300D400, ② BP200D600, and ③ BP100D800. i) Illustration of the immobilization of the three samples which are treated with the same conditions. ii) Shape recovery process of V recorded from a side view, displaying the different recovery speeds of the three materials. The images were taken by a camera, and processed with a glowing edge effect. All the samples were bent to U shape at 60 °C and fixed a temporary shape at 23 °C. The shape recovery performed at 60 °C.

As illustrated in Figure 1, the substrate was expected to perform a programmable‐morphing process from microwells toward an aligned pattern to facilitate the differentiation behavior of NSCs. The requirement for the material to undergo a large change in structural conformation ultimately inspired us to use SMPs as the primary 4D component of the substrate. The shape memory effect provides a programmed control over both dynamic topography and dynamic internal stress, which is released automatically from the polymer as the object is transformed between different geometrical structures.^[^[qv: 22]^]^ The incorporation of topographical features and mechanical force to form a dynamic microenvironment may be the most effective approach to precisely guide stem cell differentiation toward the desired cell lineage. **Figure** **2**a shows a typical shape change process of thermoset SMPs. Chemical crosslinks in the SMPs are usually utilized to set the permanent shape, while the transition temperature (*T*_trans_), typically referring to the glass transition temperature (*T*_g_), is used to control the molecular switching segments for achieving the temporary shape. As the SMPs are heated above their *T*_trans_, the molecular switching segments are “softened” and deformation can be exerted to set the temporary shape; when the temperature is decreased below the *T*_trans_, the molecular switching segments will “freeze” to immobilize the predesigned temporary shape. The SMPs will recover their permanent shape upon returning to a temperature over *T*_trans_ since the molecular switching segments are softened again, allowing the crosslink networks to revert the structure to its original shape. During this shape‐changing process of the SMPs, the chemical structure of the polymer determines the *T*_trans_ and the transformation rate (*T*_rate_). Specifically, an appropriate ratio of stiff segments and flexible segments, and moderate crosslinking density are desired to meet various demands for different *T*_trans_ and *T*_rate_. To mimic the temporal conditions of native neural development, a biomimetic neural tissue culture substrate should perform a two‐week self‐morphing transformation at 37 °C (physiological temperature or cell culture temperature) to dynamically replicate the NSC differentiation behavior.^[^[qv: 16]^]^ Thus, by simply changing the environmental temperature, the topography can be changed dynamically and in turn a time‐dependent local force can be generated automatically on these substrates, which can be further used to direct stem cell behavior and function.

**Figure 3:**
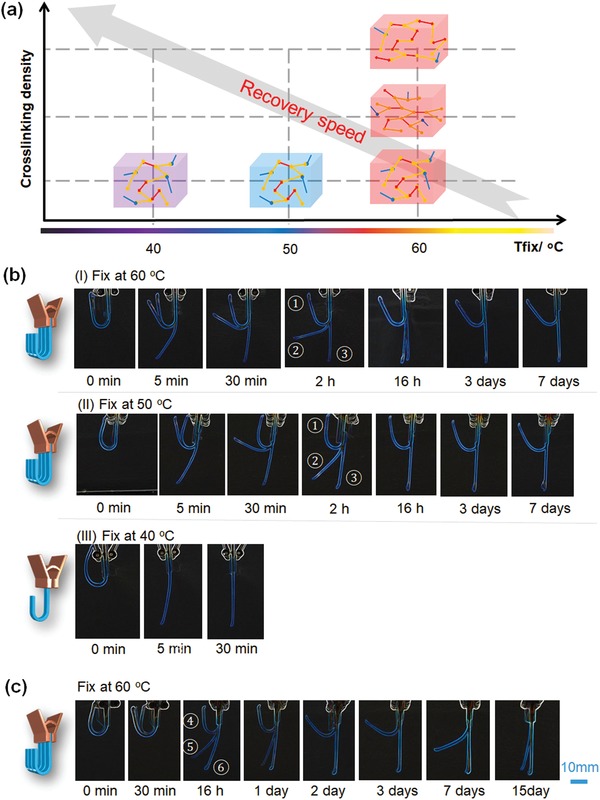
Shape memory behavior of the synthesized polymeric materials when performing the shape fix at different temperatures and the shape recovery at 37 °C. a) Illustration of the recovery speed of shape memory polymers when varying the fixing temperature and crosslinking density. b) Shape recovery process of shape memory polymers: ① sample BP300D400; ② sample BP200D600; ③ sample BP100D800, displaying the different recovery speeds at different fixing temperatures, where I) fixing the temporary shape at 60 °C and recovering at 37 °C, II) fixing at 50 °C and recovering at 37 °C, and III) fixing at 40 °C and recovering at 37 °C. c) Shape recovery process of shape memory polymers: ④ sample BP275D450; ⑤ sample BP250D500; ⑥ sample BP225D550, which were fixed at 60 °C and recovered at 37 °C.

Initially, we explored the shape memory properties of our synthesized materials by manipulating the components and the processing parameters of the SMPs. Five samples were synthesized with the formulations listed in the first five rows of Table S1 and Figure S1 (Supporting Information) shows the corresponding DSC results. The *T*_g_s of all samples were observed, as they underwent physical transformations from the glassy state to the rubbery state. The endothermic process of our curves illustrates that heat flows into the samples as a result of glass transition. In our design, the amount of the stiff epoxy monomer (bisphenol A diglycidyl ether, B) was kept constant, while the ratio of flexible aliphatic crosslinker ((poly(propylene glycol) bis(2‐aminopropyl) ether, P) or crosslinking modulator (decylamine, D) was changed to adjust the crosslinking density. To ensure the completion of the reaction of the epoxy, the concentration of the monoamine crosslinking modulator was controlled to adjust the crosslinking degree and to keep the total amine content equal to the epoxy content. To perform the shape transformation at 37 °C, a sample with a *T*_g_ around 37 °C was designed, so that the fixing temperature and the recovery temperature were set below and above 37 °C, respectively. Among these samples, BP300D400, BP200D600, and BP100D800 exhibited *T*_g_s at 46, 40, and 25 °C, respectively. Here, BP300D400 represents that P is 0.00300 mole and D is 0.00400 mole when B content is kept at a constant 0.01 moles (the details are shown in Table S1 in the Supporting Information). All of these samples were able to be softened and deformed into a U shape at ≈60 °C, and could be fixed in a temporary shape at room temperature (average ≈23 °C) (Figure 2b‐i). As shown in Figure 2b‐ii, the samples displayed different *T*_rates_ at 60 °C in their recovery processes, due to their different *T*_g_ and crosslinking density. Generally, the recovery speed of these materials is determined by the switching phase. Thus, samples with a lower *T*_g_ and a higher crosslinking density contribute to a faster recovery speed (**Figure** **3**a). It can be demonstrated that the switching phase of the sample which has a lower *T*_g_ is softer at the recovery temperature, which leads to faster shape recovery. Additionally, a higher crosslinking density increased the responsive efficiency of the switching phase.

Although these samples demonstrated a typical shape recovery process, they cannot be directly used as cell culture matrices to modulate NSCs differentiation behaviors, due to their high recovery temperature and the fast recovery speeds. Therefore, we further investigated the recovery behavior of these samples at 37 °C (Figure 3b). As expected, all samples exhibited a significantly slower recovery speed than that at 60 °C (Figure 3b‐I). BP200D600 and BP100D800 fully recovered their original shape at three days, while BP300D400 could not completely recover at 15 days. For the neural development study, BP300D400 is too slow while BP200D600 is too fast. Additionally, another factor that influences recovery speed is the deformation temperature. As illustrated in Figure 3a, the switching segments in the SMP are much stiffer at a lower temperature, so more energy is stored when deformed, leading to faster shape recovery. When the samples were fixed at 50 °C, the recovery speeds of samples were faster (Figure 3b‐II). Moreover, when the recovery temperature was set at 40 °C, only BP100D800 fully recovered within 30 min, while BP300D400 and BP200D600 were unable to recover their original shapes due to their high *T*_g_s (Figure 3b‐III). We can conclude that the effects of SMP formulation or chemical structure on shape change are much higher than that of the deformation temperature. Thus, we further developed three more samples as listed in the last three rows in Table S1 (Supporting Information), where the ratio between stiff and soft monomers was ranged from 3:4 (BP300D400) to 1:3 (BP200D600) based on the previous results in Figure 3b. Considering that the deformation temperature does not have a remarkable effect on the shape recovery, we performed the deformation temperature at 60 °C, and investigated the recovery process at 37 °C. As shown in Figure 3c, BP275D450 showed a recovery duration up to 2 weeks while BP250D500 and BP225D550 fully recovered their original conformations within two days. Therefore, we expected that the shape change achieved by BP275D450 could provide an appropriate timeframe for NSC aggregation and the subsequent neural cell outmigration. Moreover, the actual transformation time of BP275D450 can also be controlled by changing the deformation degree or pattern to obtain a faster recovery speed (from 3 days to 15 days).

### 2.2 Fabrication of Microwell Arrays and Dynamic Cell Aggregation

**Figure 4:**
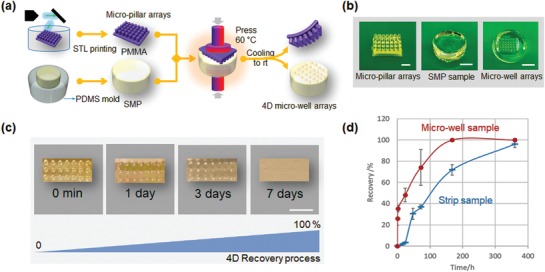
Fabrication and characterization of microwell arrays. a) Illustration of the fabrication of microwell arrays. b) Photoimages of STL printed micropillar arrays with 800 × 800 µm pillar size (scale bar, 2.5 mm), SMP sample (scale bar, 5 mm), and 4D microwell arrays with 800 × 800 µm well size (scale bar, 5 mm). c) Shape transformation process from microwell to flat with the sample having an 800 × 800 µm well size. When incubated in water bath at 37 °C, the microwell arrays were gradually transformed, which were still visible at three days, and almost restored its original flat surface after seven days. d) Dynamic recovery process of the strip sample and microwell array sample demonstrated by the relationship between recovery degree and incubation time.

To investigate the transformative ability of patterning to promote the NSC aggregation, we determined the shape recovery properties of BP275D450 in the transformation from microwells to a flat surface. As shown in **Figure 4**a,b, we created microwell arrays on SMP samples by imprinting stereolithography (STL) printed micropillar poly(methyl methacrylate) (PMMA) arrays via a thermomechanical reprogramming process. Since PMMA has a much higher *T*_g_ (≈105 °C) than BP275D450, the microwell arrays were successfully generated after imprinting at 60 °C, and the temporary microwell arrays were immediately fixed after cooling to room temperature. The shape transformation process from microwells to a flat surface is illustrated in Figure 4c with the sample having an 800 × 800 µm well size. When incubated at 37 °C, the microwell arrays were gradually transformed, and were still visible after three days. By day 7, the SMP samples had completely restored their original flat surfaces. The dynamic recovery process of the U shape strip sample and microwell arrays are compared in Figure 4d. The microwell arrays showed a faster recovery speed than the strip samples, which might be explained by the higher energy storage or lower deformation degree during the microwell deformation. Taking the sample fidelity and operation accessibility into consideration, we prepared all the 4D culture substrates with a thickness of 6 mm to generate the microwell arrays with a depth up to a maximum of 800 µm.

**Figure 5:**
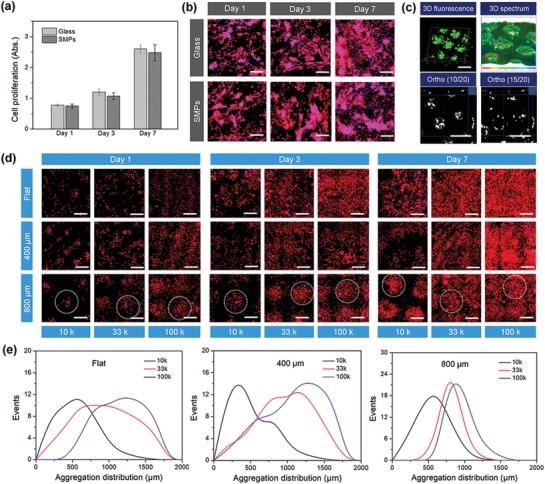
Biocompatibility of the synthesized polymeric materials and NSC aggregation behavior. a) NSC proliferation on SMP and glass samples after 1, 3, and 7 days of culture. There is no significant difference among these samples (N.S.), suggesting our SMPs exhibited an excellent cytocompatibility in vitro. b) NSC morphology on SMP and glass samples after 1, 3, and 7 days of culture, where F‐actin is colored red and the nucleus is colored blue. Scale bar is 200 µm. c) 3D fluorescent images and image analysis of GFP‐NSCs when cells were seeded in the 4D microwell arrays. The 3D spectrum showed the fluorescent intensity distribution of aggregates when NSC grew in the microwell arrays. The ortho (10/20) and (15/20) images demonstrated the aggregation morphology of NSCs at the different *Z* position of the microwell arrays. The scale bar is 1 mm. d) Fluorescent images of NSCs with different cell densities on different culture substrates (flat, 400 µm, and 800 µm microwells) after 1, 3, and 7 days of culture. Dotted circles indicate the NSC aggregation in the microwell region. The scale bar is 600 µm. e) Quantitative analysis of NSC size distribution by fluorescence aggregation at 3 days.

In order to evaluate the cytocompatibility of our SMPs, we performed a standard cell proliferation assay (CCK‐8 assay). The results showed that our SMP substrate without any surface treatment or modification (protein coating) had excellent cell growth after two weeks of culture, similar to the glass substrate (control) (**Figure** **5**a). Additionally, the cell morphology of the seeded NSCs was also investigated using F‐actin staining. Fluorescence images illustrated that the morphology of NSC adhesion on the nontreated SMP substrate was comparable with the glass substrate (Figure 5b). Having established that the SMP substrates could promote cell survival and growth, we sought to explore time‐dependent neural development and the effect of 4D transformation on the neural differentiation of NSCs. To this end, we first investigated the NSC aggregating behavior in the microwell arrays during the first three days of culture. After 3 days of culture, the aggregation of GFP‐NSCs was studied by 3D confocal scanning (Figure 5c). We further analyzed the images to better observe the aggregation feature of the NSCs. The 3D spectrum showed the fluorescent intensity distribution of the aggregates when the NSCs grew in the microwell arrays. After the 3D images were split into 20 slides of 2D images, the ortho (10/20) and (15/20) images displayed the aggregation morphology of the NSCs at the different Z positions of the microwell arrays.

After which, we further explored the effects of cell number and microwell size on the aggregating behaviors of NSCs. The microwell arrays with two feature diameters (400 and 800 µm) were imprinted using STL‐printed micropillar PMMA arrays, and a flat sample served as a control. The NSCs with different cell densities (1 × 10^4^, 3.3 × 10^4^, 1 × 10^5^ cells mL^−1^) were seeded into the patterned microwells and were cultured to confluence after seven days of 4D transformation, in order to generate optimal size‐controlled aggregation. The fluorescence images showed the formation of NSC aggregates on different culture substrates, as is illustrated in Figure 5d. The conventional aggregate formation on the flat substrate resulted in a heterogeneous distribution of cell clumps of various sizes. By the first day of culture, the 400 µm (small size) microwells yielded much smaller cell aggregates, however, the NSC density in these small wells was unable to form the uniform and dense aggregates during the 4D transformation of the substrate. In contrast, the 800 µm (large size) microwells generated uniform, compact NSC aggregates of controlled size, as we designed. Moreover, after seven days of culture, higher cell densities (both 3.3 × 10^4^ and 1 × 10^5^ cells mL^−1^) exhibited excellent cell confluence or monolayers around loose aggregates. The quantitative analysis of NSC size distribution was also performed by fluorescence aggregation to determine the optimal cell seeding number at 3 days of culture (Figure 5e). Results showed that when the cell density was higher than 3.3 × 10^4^ cells mL^−1^, uniform NSC aggregates were formed at three days, and further continue to confluence at seven days. This typical process fully replicated the NSC behaviors (morphology and biological features) of the first four stages of in vitro neural differentiation including noninduced, initial changes, aggregation, and outmigration.^[^[qv: 16]^]^ The data suggested that both cell density and microwell size influenced NSC aggregation behaviors, which is expected to affect the subsequent differentiation pathways.

### 2.3 Fabrication of 4D Culture Neural Substrate and Dynamic Axon Guidance

**Figure 6:**
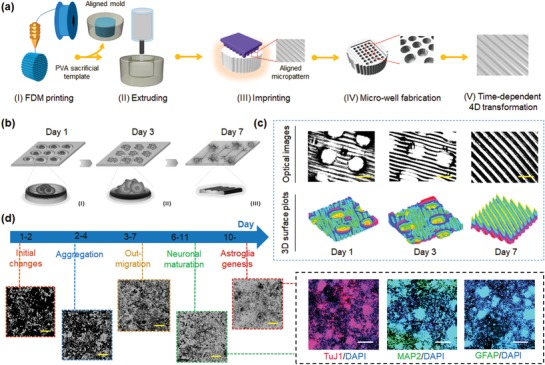
Fabrication and characterization of 4D aligned/microwell arrays on SMPs. a) Illustration of the fabrication of microwell arrays and their time‐dependent 4D transformation. b) Illustration of time‐dependent cell patterning process during 7 days of culture. I) Reprogrammed microwell arrays and cell seeding at day 1. II) 4D shape recovery and NSC aggregation at day 3. III) Original aligned patterns and axonal alignment of the differentiating NSCs at day 7. c) Optical images and 3D surface plots of 4D SMP substrates changing from microwell arrays to aligned pattern (microgrooves) at 1, 3, and 7 days. The scale bar is 800 µm. d) Illustration of time‐dependent differentiation behavior of NSCs, demonstrating the different stages of RA induced in vitro neural differentiation of NSCs (microscopic images), and the biological features (the expression of neurogenic markers) in the differentiated NSCs after 14 days of culture. Neuronal marker: TuJ1, mature neuronal markers MAP2, and astrocytes marker GFAP. Scale bar is 200 µm.

Several studies have demonstrated that the specific response of neuronal cells to topographical cues can result in axon guidance and the growth of transplanted neurons to enhance therapeutic effects for nerve injury repair.^[^[qv: 28,29]^]^ Therefore, in order to precisely control the directional growth and influence the neuronal differentiation of NSCs, the aligned topographical features were further created to direct the formation of highly aligned axons from the differentiating NSCs. Based on previous studies, it has been demonstrated that surfaces with micro‐ or nanoscale topographical patterns were able to control NSC alignment and improve neurogenesis.^[^[qv: 29,30]^]^ As shown in **Figure** **6**a, highly aligned micropatterns with a width of 100 µm were prepared on the surface of the samples. As such, a “key‐lock copied” template strategy that incorporates a 3D fused deposition modeling (FDM) printed poly(vinyl alcohol) (PVA) sacrificial mold and polydimethylsiloxane (PDMS) microgrooves was utilized to create an aligned micropattern with a width of 100 µm, which is further described in the Experimental Section. After which, the microwell arrays were created on the aligned micropatterned sample using the aforementioned “thermomechanical imprint” method. The resultant 4D culture substrate that integrated “temporary” microwell arrays and “programmable” aligned patterns were used to facilitate the time‐dependent differentiation behavior of NSCs (Figure 6b). As shown in Figure 6c, optical images and 3D surface plots displayed the 4D transformation behavior of the culture substrates with time. The images confirmed that the substrate surface pattern changed from “temporary” microwell arrays to “programmable” aligned patterns.

**Figure 7:**
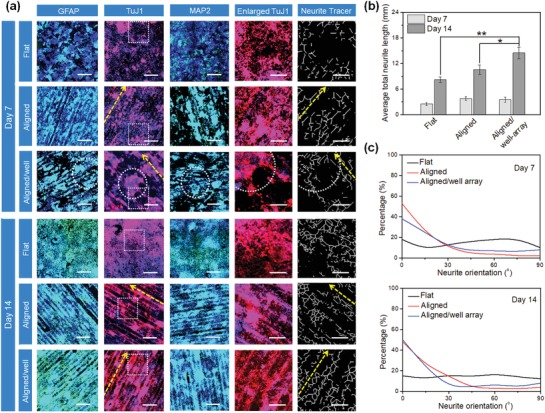
NSC differentiation studies on 4D aligned/microwell arrays on SMPs. a) Immunofluorescent images of NSC differentiation on 4D Aligned/microwell arrays compared to flat samples and samples with alignment only, after culturing in differentiation medium for 2 weeks. GFAP and MAP2 are colored green, TuJ1 is colored red, and nuclei are colored blue. The scale bar is 500 µm. The dotted squares in TuJ1 images indicate the enlarged area, which is used to generate the images of neurite tracer (scale bar is 200 µm). The dotted circles indicate the NSC aggregation in microwell. The yellow dotted arrows show the direction of aligned patterns (microgrooves). b) Quantification of total neurite length of neural differentiation 4D aligned/microwell arrays when compared to other corresponding groups at 7 days and 14 days. Data are mean ± standard deviation; *n* = 9; *p < 0.05. c) The neurite direction of the microgrooves was set as an angle of 0° (horizontal direction was set as 0° for the flat control samples). The neurite major axis with respect to the direction of the microgrooves (or horizontal direction for flat control) was defined as the neurite orientation. The neurites were considered to be aligned if their angles fell into ±20° from the original benchmark.

Figure 6d shows the different stages of retinoic acid (RA) induced in vitro neural differentiation of NSCs, and the biological features (the expression of neurogenic markers) in the differentiated NSCs. As mentioned above, NSCs tend to form aggregates early in the differentiation process, while neurite extension and elongation tends to occur in the later stages of development.^[^[qv: 16]^]^ We thus hypothesized that gradually changing 4D SMP substrates could be an excellent platform to enhance the differentiation of NSCs into neurons and glial cells, and further control axonal alignment of the differentiating NSCs. Our immunostaining data demonstrated that the developing NSC aggregates became clearly out‐migrated while the well‐array gradually disappeared after seven days of differentiation. Therein, the majority of the aligned axons from the differentiated NSCs were identified by the expression of the neuron‐specific marker class‐III β‐tubulin (TuJ1) on the 4D aligned/well‐array substrates (**Figure** **7**a). The presence of the mature neuronal marker microtubule‐associated protein 2 (MAP2) and astrocytes marker glial fibrillary acidic protein (GFAP) were characterized to confirm neuronal maturation and astroglia genesis, respectively. Compared to that of the flat and 3D aligned substrates (microgrooves), the elevated expression levels of these specific neuronal markers demonstrated that the 4D aligned/well‐array substrates accelerated the differentiation of NSCs. It can be deduced that the well‐arrays improved the formation of NSC aggregates, and thus led to earlier neural differentiation. Interestingly, significant interconnected neuronal aggregates and astroglia genesis were also observed after 14 days of culture. Moreover, the quantification of total neurite length of neural differentiation on the 4D aligned/microwell arrays was also evaluated after 7 days and 14 days of culture (Figure 7b). The aligned/microwell arrays exhibited the longest neurite length when compared to the other groups. After two weeks of differentiation (with the continuous recovery of originally aligned patterning), it was observed that the differentiating NSCs showed excellent axonal alignment on the 4D aligned/well‐array substrates (Figure 7c). Results demonstrated that the engineered microenvironment consisting of 4D dynamic transformation and microtopographical feature provides instructive physical cues that lead to enhanced neural differentiation of NSCs along with significant axonal alignment.

**Figure 8:**
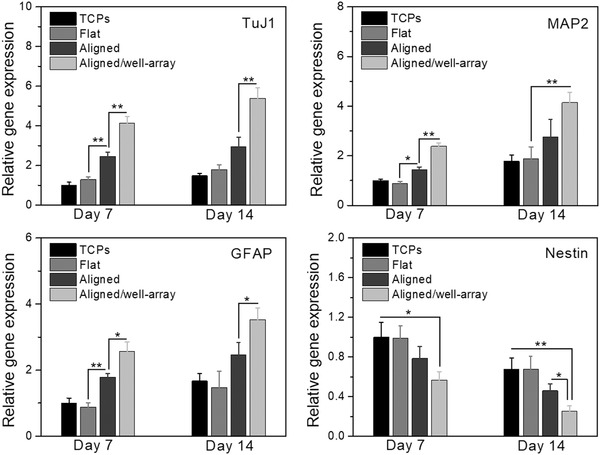
Gene expressions of NSCs on 4D aligned/microwell arrays when compared to TCPs, flat samples, and samples with aligned only for 2 weeks of culture. The data were normalized to the expression levels of cells on TCPs. Data are reported as mean ± standard deviation, *n* = 6, *p < 0.05, **p < 0.01.

Next, in order to quantify the expression levels of these neuronal markers, we further performed real‐time quantitative polymerase chain reaction (rt‐qPCR) analyses of neural differentiation of NSCs on 4D aligned/well‐array substrates and compared them to NSCs differentiated on only aligned, flat, and tissue culture plate (TCP) substrates. Generally, the gene expression of TuJ1, MAP2, and GFAP within differentiating NSCs increased with time, whereas the undifferentiated NSC marker Nestin decreased for all culture substrates. The expression levels of neuronal and axonal markers were upregulated on aligned and 4D aligned/well‐array substrates compared to the control TCP and flat substrates. Moreover, we found that the differentiation of NSCs on the 4D aligned/well‐array substrates showed the highest expression levels for the neuronal markers (TuJ1 and MAP2) and the astrocyte marker (GFAP) (**Figure** **8**). It can be concluded that the combined effect of having microwells and an aligned pattern on a single substrate yields significantly enhanced neural differentiation and remarkable alignment of differentiated NSCs. Therefore, it has been demonstrated the dynamic 4D effect may better mimic the special growth microenvironments of neural tissue, and provide a potential method for modulating different developmental stages of NSCs, and can thus hasten the functional recovery of injured neural tissues.

In this study, the results indicate that our 4D self‐morphing substrate provides a cutting‐edge technology to manipulate cell functions and fates, and can thus serve as the platform by which to develop a new generation of commercially available scaffolds and devices. This concept of dynamic culture can be extended to other bioengineering systems. These novel 4D tissue culturing substrates can be used to mimic the progression of specific diseases, which can thereby advance life science research as a whole. In addition to functioning as a platform by which to make dynamic cell culture substrates, the 4D SMPs also have utility for application in tissue regeneration, biomechanics research, bio‐robotics, and cancer therapy.

## 3 Conclusion

A 4D dynamic culture substrate was developed for enhancing the growth and differentiation of neural stem cells. The smart 4D shape transformation was able to facilitate neural tissue development from cellular aggregates to highly aligned axons, which replicates the physiological characteristics of NSC‐derived neural development. It is expected that this project will have a far‐reaching impact on not only neural tissue regeneration, but also other bioengineered tissues.

## 4 Experimental Section

##### Preparation and Characterization of Shape Memory Polymers

Poly(propylene glycol) bis(2‐aminopropyl ether), bisphenol A diglycidyl ether, and decylamine were mixed homogeneously in a glass beaker at room temperature. The mixture was then centrifuged at 1500 rpm for 3 min to remove bubbles. The ratio of the components was varied to determine the properties of the final constructs. The 4D ink was precured at 100 °C for 1.5 h, and finally cured at 135 °C for another 1.5 h. The shape memory properties of the solidified materials were characterized according to our previous methods.^[^[qv: 26,31]^]^ Briefly, the sample strips were folded 180° into a “U” shape at different temperatures with an inner radius of 10 mm, and were kept in that conformation for 10 min. The strips were then immediately cooled to room temperature for an additional 10 min in order to obtain a temporary shape. The “U” shaped strips were then immersed in the water bath at 37 °C to recover the permanent shape. The dynamic shape change (recovery speed) of different models was recorded with a PowerShot ELPH 360HS Cannon camera. The images were processed using glowing edges filter to enhance the object's border.

##### Fabrication of Programmable Culture Substrates

Three different printing techniques, including FDM, extrusion, and STL, were used to fabricate the programmable culture matrices. First, a cylinder sacrificial mold with a diameter of 12 mm and a height of 8 mm was designed with the software Autodesk123D (Autodesk Inc, USA), and the stl. formatted file was then loaded into the software Slic3r which is licensed under the GNU Affero General Public License, version 3. The infill density, the printing speed, and the layer height were assigned in Slic3r. The predesigned structures were then printed via a Solidoodle 3D FDM printer platform with a nozzle size of 100 µm. Open‐source software (Prontrface) was utilized to control the three stepper motors with an effective resolution of 100 µm in the *x*‐ and *y*‐axis, and a minimum layer height of 50 µm in the *z*‐axis. A PVA filament used for 3D printing was obtained from Matter Hackers (USA). The PVA filament with a diameter of 1.75 mm was used as the sacrificial mold material, and the printing temperature was set at 190 °C. The PVA sacrificial mold was printed with a layer height of 100 µm to generate the aligned surface structure. The mold was then coated with PDMS and cured at 70 °C for 2 h in order to obtain PDMS microgrooves with a width of 100 µm. Next, preset amounts of the 4D ink materials were then extruded into the PDMS mold, and were then cured using the process described above. After the samples had cooled to room temperature, the aligned micropatterned scaffolds were taken out of the oven and were ready for additional operations.

Finally, microwells were generated on our flat and aligned scaffold to improve the NSC spheroid formation at an early stage of neural development. A cubic mold 9 mm (*L*) × 6 mm (*W*) × 2 mm (*H*) with micropillars (800 and 400 µm in diameter) was designed with the software Autodesk 123D, and saved as an stl. format file. The models were uploaded into a desktop STL printer (Formlabs, USA) to slice the digital model into layers for printing. Two different diameters of micropillar templates, 800 and 400 µm, were printed with PMMA resin for the fabrication of hard molds. The speed of operation was set to 25 mm h^−1^, the *XY* resolution was 50 × 50 µm, and the layer height was 50 µm. After printing, a thermomechanical reprogramming process was performed to create the temporary microwells on our 4D scaffolds as follows: the 4D scaffolds were imprinted by a micropillar mold with a clamp at 60 °C for 10 min, and then moved to ice water for 10 min. Additionally, the 4D transformation behavior of the culture substrate as a function of time was captured by optical microscopy, and 3D surface plots were created with AmScope 3.7 software.

##### Cell Culture, Proliferation, and Morphology

NSCs cloned from mouse neuroectoderm (NE‐4C) were purchased from American Type Culture Collection (ATCC). NSCs (passage no. 3–6) were cultured in Eagle's minimum essential medium (ATCC) supplemented with 5% fetal bovine serum (FBS), 1% (v/v) l‐glutamine, and 1% penicillin/streptomycin solution, under standard cell culture conditions (37 °C, a humidified, 5% CO_2_/95% air environment). NSCs were seeded on glass and SMPs (without any surface treatment or modification) at a density of 5 × 10^4^ cells mL^−1^ and continuously cultured for 1, 3, and 7 days. At the predetermined time interval, culture medium containing 10% CCK‐8 solution (Dojindo, Japan) was added and incubated for 2 h. 200 µL of the medium was transferred into a 96‐well plate, and the absorbance of the incubated solution at a wavelength of 450 nm was quantified by a spectrophotometer (Thermo, USA). The NSC morphology was evaluated by F‐actin staining. At each predetermined time, all samples were fixed with 10% formalin for 15 min and then permeabilized with 0.2% Triton‐100 for 10 min. The samples were then stained with a Texas Red‐X phalloidin solution (1:100) to stain the cells' cytoskeleton for 30 min, followed by 4′,6‐diamidino‐2‐phenylindole (DAPI) (1:1000) solution to stain the cells' nuclei for another 5 min. The images were observed using laser confocal microscopy (Carl Zeiss LSM 710).

##### Cell Aggregation Evaluation

The green fluorescent protein transfected NSCs (GFP‐NSCs, NE‐GFP‐4C, ATCC) were seeded on the microwell arrays of the 4D substrate at a density of 5 × 10^4^ cells mL^−1^ and cultured for 3 days. The 3D fluorescent images were taken by confocal microscopy and analyzed by Zen software (Zeiss). Additionally, in order to optimize the formation of NSC spheroids, NSCs were seeded on the scaffolds at different densities of 1 × 10^4^, 3.3 × 10^4^, 1 × 10^5^ cells cm^−2^, and cultured for 7 days under standard cell culture conditions. At each predetermined time, the cells were fixed with 10% formalin for 15 min and permeabilized in 0.1% Triton X‐100 for 10 min. The cells were stained with Texas red fluorescent dye for 30 min and then DAPI blue fluorescent dye for 5 min to observe NSC aggregation on the scaffolds. The double‐stained samples were imaged on the confocal microscope. The aggregation distribution of NSCs at 3 days was quantified by Image J analysis software (National Institutes of Health). After setting a scale bar, the distribution area was adjusted using threshold settings, and then was measured. Six visible areas were randomly selected for statistical analysis on each sample; there were five samples in each group.

##### Neurogenic Differentiation

The neurogenic differentiation of NSCs was performed using our reported method with modifications. The NSCs were seeded on various scaffolds at 3 × 10^4^ cells cm^−2^ and maintained in the growth medium for 24 h. To induce the neurogenic differentiation, the samples were cultured in the neurogenic medium, which consisted of the growth medium supplemented 10^−6^ m retinoic acid (RA), and the medium was exchanged every other day.

##### Immunofluorescence Staining

Neurogenic differentiation of NSCs was identified using immunofluorescence staining. After incubation with neurogenic differentiation medium for 7 and 14 days, the cells were fixed with 10% formalin for 15 min, and were then treated with 0.1% Triton X‐100 for 10 min. Then the samples were incubated with a blocking solution (containing 1% bovine serum albumin (BSA), 0.1% Tween 20 and 0.3 m glycine in PBS) for 2 h. The first primary antibodies of mouse anti‐TuJ1 (1:1000), rabbit anti‐GFAP antibody (1:500) and rabbit anti‐MAP2 antibody (1:500) were gently mixed with samples overnight at 4 °C. Next, the secondary antibodies of goat antimouse Alexa Fluor 594 (1:1000) and goat anti‐rabbit Alexa Fluor 488 (1:1000) were incubated with samples in the dark for 2 h at room temperature, followed by DAPI (1:1000) solution incubation for 5 min. The immunofluorescence images were taken using confocal microscopy. The average total neurite length and orientation were quantified by Image J analysis software (NeuriteTracer). Three visible areas were randomly selected for quantifying statistical analysis on each sample; there were three samples in each group. The direction of the microgrooves was set as an angle of 0° (horizontal direction was set as 0° for the flat control samples). The neurite major axis with respect to the direction of the microgrooves (or horizontal direction for flat control) was defined as the neurite orientation. The neurites were considered to be aligned if their angles fell into ±20° from the original benchmark.

##### Real‐Time Quantitative Polymerase Chain Reaction

The neurogenic gene expression of all samples, including neuron‐specific class III β‐tubulin (TuJ1), MAP2, GFAP, and Nestin, were analyzed by the rt‐qPCR assay. Briefly, the total RNA content was extracted from the samples using Trizol reagent (Life Technologies). The RNA quality and concentration were determined from the absorbance at 260 and 280 nm with a microplate reader. RNA samples were reverse‐transcribed to cDNA using a Prime Script RT reagent Kit (TaKaRa). RT‐PCR was then performed on a CFX384 Real‐Time System (BIORAD) by using SYBR Premix Ex Taq (TaKaRa) according to the manufacturer's protocol. The gene expression level of target genes was normalized against the housekeeping gene glyceraldehyde 3‐phosphate dehydrogenase (GAPDH). The relative gene expression of the samples was normalized against the control group to obtain relative gene expression fold values and calculated via the 2‐delta delta (2^−ΔΔCt^) cycle‐threshold method. Primer sequences are as follows: TUJ1, forward primer 5′‐AGCTGTTCAAACGCATCTCG‐3′ and reverse primer 5′‐GACACCAGGTCATTCATGTTGC‐3′; MAP2, forward primer 5′‐TTCTCCACTGTGGCTGTTTG‐3′ and reverse primer 5′‐GAGCCTGTTTGTAGACTGGAAGA‐3′; GFAP, forward primer 5′‐CCTTCCTTCCCTGGTTTTCT‐3′ and reverse primer 5′‐TGCTCATCTTTCCTCTTCCC‐3′; Nestin, forward primer 5′‐GTGGCCTCTGGGATGATG‐3′ and reverse primer 5′‐TTGACCTTCCTCCCCCTC‐3′; GAPDH, forward primer 5′‐GTGGCCTCTGGGATGATG‐3′ and reverse primer 5′‐ACTCCTCAGCAACTGAGGG‐3′.

##### Statistical Analysis

The mean and standard deviation was plotted for each sample group (*n* = 6). Then, a one‐way analysis of variance (ANOVA) (*p* < 0.05) with a posthoc Tukey honestly significant difference test was performed on each set of data. The statistical significance was indicated with an asterisk. That is, samples connected with an asterisk were significantly different.

## Conflict of Interest

The authors declare no conflict of interest.

## Supporting information

Supporting Information
