# Supplementary material for: 4D Self‐Morphing Culture Substrate for Modulating Cell Differentiation
Source: Adv Sci (Weinh). 2020 Feb 18;7(6):1902403. doi: 10.1002/advs.201902403 (PMC7080541; doi:10.1002/advs.201902403)
Supplement: Supplementary file 1 — Supporting Information [file ADVS-7-1902403-s001.pdf]

Copyright WILEY-VCH Verlag GmbH & Co. KGaA, 69469 Weinheim, Germany, 2020.

Supporting Information

for *Adv. Sci.,* DOI: 10.1002/advs.201902403

4D Self-Morphing Culture Substrate for Modulating Cell Differentiation

*Shida Miao, Haitao Cui, Timothy Esworthy, Bhushan Mahadik, Se-jun Lee, Xuan Zhou, Sung Yun Hann, John P. Fisher, and Lijie Grace Zhang**

Copyright WILEY-VCH Verlag GmbH & Co. KGaA, 69469 Weinheim, Germany, 2020.

Supporting Information

4D Self-morphing Culture Substrate for Modulating Cell Differentiation

*Shida Miao, Haitao Cui, Timothy Esworthyy, Bhushan Mahadik, Se-jun Lee, Xuan Zhou, Sung Yun Hann, John P. Fisher, and Lijie Grace Zhang**

Table S1. The components and formulae of the synthesized polymeric materials.

NO.	Code	Bisphenol A diglycidyl ether (B)		Poly(propylene glycol) bis(2-aminopropyl ether) (P)		Decylamine (D)	
		/g	/mol	/g	/mol	/g	/mol
1	BP500D000	3.40	0.010	1.15	0.00500	0.00	0.00000
2	BP400D200	3.40	0.010	0.92	0.00400	0.33	0.00200
3	BP300D400	3.40	0.010	0.69	0.00300	0.66	0.00400
4	BP200D600	3.40	0.010	0.46	0.00200	0.99	0.00600
5	BP100D800	3.40	0.010	0.23	0.00100	1.32	0.00800
6	BP275D450	3.40	0.010	0.63	0.00275	0.75	0.00450
7	BP250D500	3.40	0.010	0.58	0.00250	0.83	0.00500
8	BP225D550	3.40	0.010	0.52	0.00225	0.91	0.00550

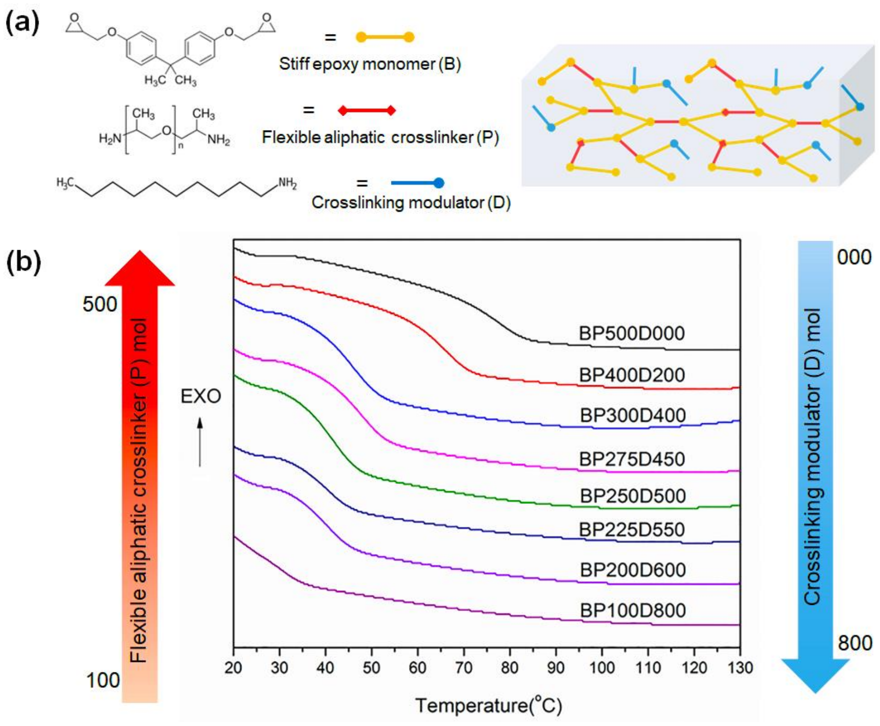

Figure S1. Synthesis and characterization of synthesized polymeric materials. (a) Chemical structure of shape memory polymers (SMPs) including a stiff epoxy monomer (B), a flexible aliphatic crosslinker (P) and a crosslinking modulator (D). (b) DSC curves of synthesized polymeric materials. The EXO with an arrow shows the exothermic process.
